# Statistical model building: Background “knowledge” based on inappropriate preselection causes misspecification

**DOI:** 10.1186/s12874-021-01373-z

**Published:** 2021-09-29

**Authors:** Lorena Hafermann, Heiko Becher, Carolin Herrmann, Nadja Klein, Georg Heinze, Geraldine Rauch

**Affiliations:** 1 Charité – Universitätsmedizin Berlin, corporate member of Freie Universität Berlin and Humboldt-Universität zu Berlin, Institute of Biometry and Clinical Epidemiology, Charitéplatz 1, Berlin, 10117 Germany; 2 Institute of Medical Biometry and Epidemiology, University Hospital Hamburg-Eppendorf, Martinistraße 52, Hamburg, 20246 Germany; 3 Humboldt Universität zu Berlin, School of Business and Economics, Unter den Linden 6, Berlin, 10099 Germany; 4 Section for Clinical Biometrics, Center for Medical Statistics, Informatics and Intelligent Systems, Medical University of Vienna, Spitalgasse 23, Vienna, 1090 Austria

**Keywords:** Background knowledge, Univariable selection, Backward elimination, Variable selection, Regression model, Simulation study, Need for more data sharing

## Abstract

**Background:**

Statistical model building requires selection of variables for a model depending on the model’s aim. In descriptive and explanatory models, a common recommendation often met in the literature is to include all variables in the model which are assumed or known to be associated with the outcome independent of their identification with data driven selection procedures. An open question is, how reliable this assumed “background knowledge” truly is. In fact, “known” predictors might be findings from preceding studies which may also have employed inappropriate model building strategies.

**Methods:**

We conducted a simulation study assessing the influence of treating variables as “known predictors” in model building when in fact this knowledge resulting from preceding studies might be insufficient. Within randomly generated preceding study data sets, model building with variable selection was conducted. A variable was subsequently considered as a “known” predictor if a predefined number of preceding studies identified it as relevant.

**Results:**

Even if several preceding studies identified a variable as a “true” predictor, this classification is often false positive. Moreover, variables not identified might still be truly predictive. This especially holds true if the preceding studies employed inappropriate selection methods such as univariable selection.

**Conclusions:**

The source of “background knowledge” should be evaluated with care. Knowledge generated on preceding studies can cause misspecification.

**Supplementary Information:**

The online version contains supplementary material available at (10.1186/s12874-021-01373-z).

## Background

Statistical regression models play an important role in epidemiological and medical research. The scientific aims behind those models should thereby carefully be differentiated. While explanatory models should identify causal relations and factors explaining differences in outcomes, predictive models aim at predicting an outcome variable with minimal prediction error, and descriptive models ideally capture the main associations of independent variables and outcome [1]. In many applications, several aims might also be combined. In any case, consideration of the aim of model building is essential for choosing the set of independent variables for the model, as the interpretation of coefficients of the model changes with the selected companion variables [2, 3]. In this work, we focus on the descriptive and the predictive perspective as the theory for identifying causal relations goes far beyond classical variable selection techniques [4–6].

Variable selection is an essential aspect of model building in epidemiological and medical studies. Whenever the number of candidate predictors seems too large for a meaningful interpretation or for a reliable prediction, the question is how to separate the truly predictive variables from the non-predictive ones and how assumed background knowledge influences this procedure. Many procedures have been proposed to automatize this step and many articles have been published addressing the performance of those procedures [2, 6–8]. As a consequence, to get an overview of the relative performance of those methods is a challenging task [9].

While general guidance on variable selection is still lacking, several articles agree on the recommendation that variable selection should always take background knowledge into account [2, 8]. This very general recommendation must of course be adapted to specific situations, for example if the study aim is to perform confounder selection, which however will not be investigated in this work. In a systematic review screening four major epidemiological journals, Walter et al. (2009) showed that 28% of the medical studies incorporated background knowledge in their analysis [10]. Ten years later, Talbot et al. updated the review and the incorporation of prior knowledge increased to 50% [11]. The importance of prior knowledge indeed seems plausible and intuitive, especially, when there is fundamental biologic evidence for a variable being an important predictor or for being causally related to the outcome. In absence of scientifically defensible assumptions, evidence may be insufficient and based on results from few or weak preceding studies only. Walter et al. explicitly state that *“Prior knowledge can be documented by referring to a study in the same population that resulted in the identification of risk factors for the outcome under study [...] or by one or more studies that identified each of the potential confounders”* [10]. The level of evidence for such assumptions is, however, rarely questioned. Often, these preceding studies are also based on some kind of model building strategy producing a more or less reliable subset of identified predictors. Such assumed background knowledge, which is then transferred to the current study, is thus uncertain. An intuitive statistical approach to deal with uncertainty is the use of Bayesian methods, however in the context of modelling background knowledge these methods are seldom applied in practice. Talbot and Massamba (2019) identified only one study out of 292 included studies which incorporated background knowledge based on a Bayesian approach [11]. Therefore, it seems current practice to either include a variable as a “known” predictor or to exclude it without considering a specific prior distribution. Such an approach comes with uncertainty, which depends on the appropriateness of variable selection in the preceding studies [12]. We may therefore ask the question which model building and variable selection techniques are most often applied in preceding studies. As several systematic reviews showed [13, 14], in many studies the method of univariable selection was used meaning that predictors were identified by evaluating unadjusted associations of candidate variables with the outcome. This method is known since long to perform badly when confounding is present [15]. Another commonly used approach, which is expected to perform better, is backward elimination [15].

The objective of this paper is to evaluate the reliability of evidence on predictor selection created by preceding studies. Thereby, our interest lies in a low-dimensional setting, meaning that the number of candidate predictors is much lower than the studies’ sample size. To mimic a situation often found in practice, we assumed that preceding studies identified predictors by univariable selection or backward elimination, and assessed the performance of different strategies to combine the evidence from several preceding studies by a simulation study.

## Methods

We considered a data generating mechanism characterized by a linear regression model. “True” predictors are characterized by a non-zero effect representing the “true data generating mechanism”. To base the below described simulation study on a realistic setting, we investigated a model resembling a real study by Sheppard et al. [16]. In there, the authors discuss that differences in blood pressure measurements occur between a measurement in a clinical environment and a measurement at home. They developed a multivariable linear regression model with the difference between diastolic blood pressure measured at home and at the clinic as the dependent variable [16]. The independent variables were age (*X*_*age*_) [*years*], sex (*X*_*sex*_) [0/1], the first reading of the clinical blood pressure (*X*_*cbp*.*first*_) [*mmhg*], the difference of the first and a follow-up reading of the clinical blood pressure (*X*_*cbp*.*change*_) [*mmhg*], the body mass index (*X*_*bmi*_) $[\frac {kg}{m^{2}}]$, the previous diagnosis of hypertension (*X*_*history*_) [0/1], the intake of antihypertensive medication (*X*_*antihyp*_) [0/1], the history of cardiovascular diseases (*X*_*cvd*_) [0/1] and the pulse pressure (*X*_*pp*_) [*mmhg*]. We assume in the following that the data is generated by the model

1 $$ \begin{aligned} Y & = 36 -0.08 \cdot X_{age} + 3.33 \cdot X_{sex} - 0.47 \cdot X_{cbp.first}\\ & + 0.31 \cdot X_{cbp.change} - 0.07 \cdot X_{bmi} - 0.03 \cdot X_{history} \\ & + 2.37 \cdot X_{antihyp} - 0.4 \cdot X_{cvd} - 0.06 \cdot X_{pp} + \epsilon,\\ & \epsilon \sim N(0,\sigma^{2}). \end{aligned}  $$

This true generating mechanism contains only the true predictors. The assumed coefficients of the above true generating mechanism were adapted from the published regression parameter estimates in the paper, but the interaction terms from the original study were excluded for the sake of simplicity. As a consequence, the intercept used in here deviates from the original publication in order to create reasonable values of the outcome. Moreover, the covariance structure of the exemplary model was chosen as reasonable as possible, but does not encode specific causal assumptions.

Two frequently applied variable selection methods are univariable selection and backward elimination with the Akaike Information Criterion (AIC). Although from a theoretical point of view, the Bayesian Information Criterion (BIC) as a model selection criteria may be preferred to identify the true underlying model [17], the AIC is more commonly applied in practice. In univariable selection, the final model includes only those predictors which were significant in univariable regressions. Backward elimination starts with the full model and iteratively cycles between identifying the least significant predictor and refitting the model without that predictor. The procedure is stopped if no predictor can be removed without increasing the AIC.

Note that although all variables in the true data generating mechanism are true predictors, the clinical relevance of the predictors and the size of the coefficients are different, so the impact of not selecting a true predictor is different as well. Therefore, we calculated the standardized regression coefficients of our data generating mechanism, which are 0.528 for *X*_*sex*_,− 0.406 for *X*_*cbp*.*first*_, 0.315 for *X*_*antihyp*_,− 0.268 for *X*_*age*_, 0.201 for *X*_*cbp*.*change*_,− 0.161 for *X*_*pp*_,− 0.093 for *X*_*bmi*_,− 0.050 for *X*_*cvd*_ and − 0.004 for *X*_*history*_. This should be kept in mind, when interpreting the simulation results below.

### Simulation and analysis

**Fig. 1:**
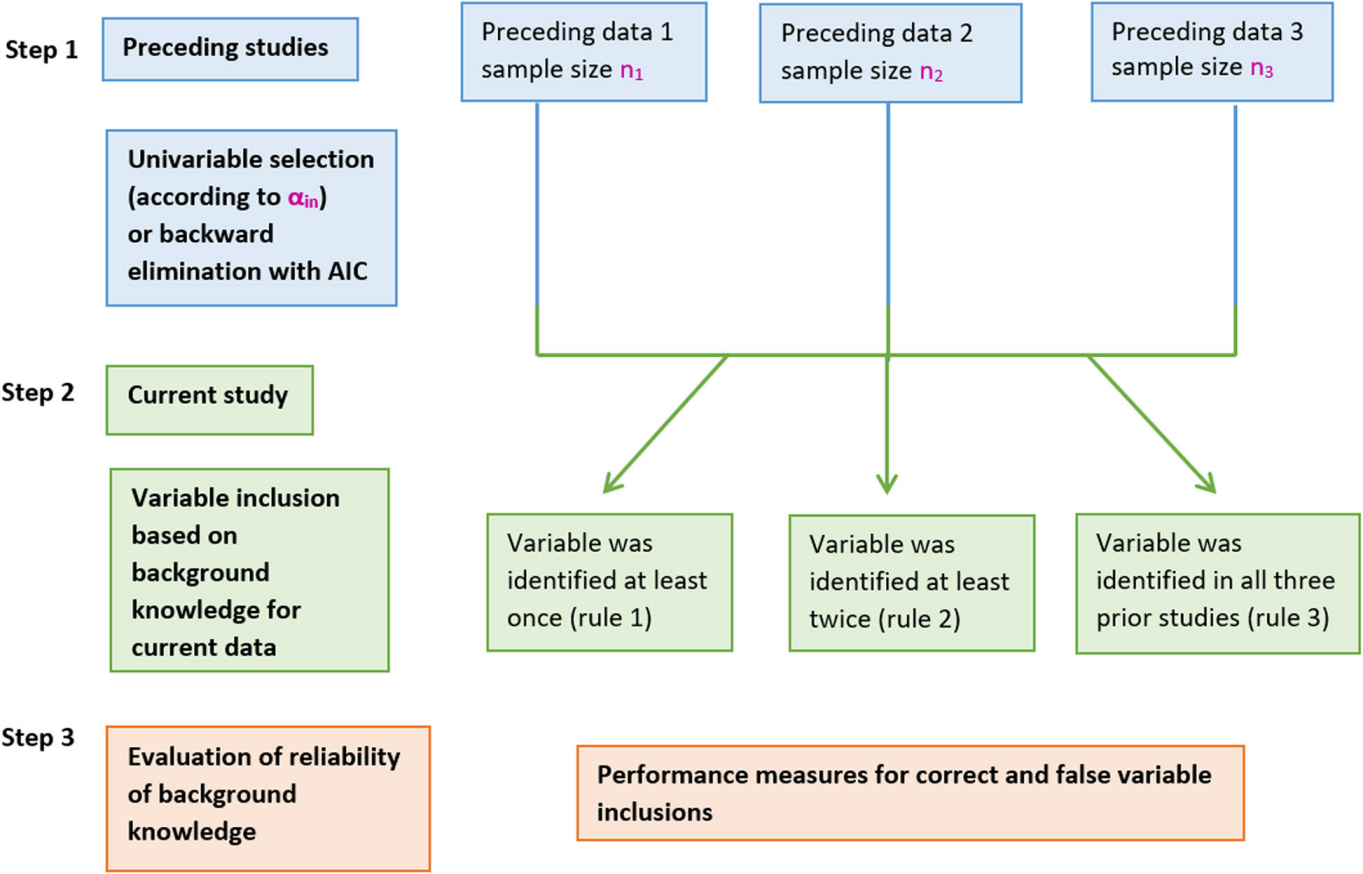
Steps of the simulation study

Our considered simulation study consisted of three steps. In the first step, data were simulated and in steps 2 and 3, the simulated data were analysed further. In step 1, three different “preceding” study data sets were generated according to the model specified above. This first step is highlighted in blue colour in Fig. 1. Subsequently, in step 2, variable selection was performed within each preceding study, and for the final model of the “present” study, a variable was considered as a “known” predictor if at least one, at least two, or all three preceding studies identified it as relevant. This part of the simulation study is graphically highlighted in green in Fig. 1. In step 3, the reliability of background knowledge based on the preceding studies was evaluated with different performance indicators. Thereby, the performance indicators assess performance aspects related to descriptive and to predictive behaviour. This third step of the simulation algorithm is highlighted in orange in Fig. 1.

In the following, the three steps are described in more detail. In the first step of the simulation study, three preceding study data sets were generated according to the true data generating mechanism, including predictor and non-predictor variables as specified in the following:

#### Step 1: data generation

**Fig. 2:**
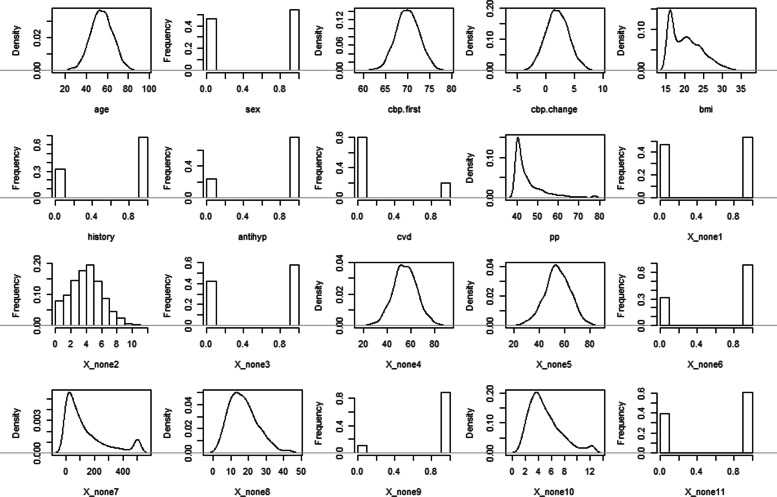
Density plots of the simulated variables

To define the candidate predictors, we additionally added a set of non-predictor variables denoted by *X*_*none*1_ to *X*_*none*11_ to the true predictor set. To simulate candidate predictors (including true predictors and non-predictor variables), we used the R-package “simdata” [18]. This package is inspired by a technical report by Binder et al. [19]. It simulates data for covariates with a predefined realistic joint distribution mimicking data from real biomedical studies. This is achieved by first drawing multivariate normal deviates with a predefinded correlation structure, and then transforming them to achieve specific realistic marginal distributions of simulated predictors and a realistic correlation structure between them. Note that the application of transformations might change the correlations. Figure 2 visualizes the respective discrete or continuous marginal densities for the simulated variables. The resulting average correlations are presented in the Supplement [Figure S1]. While the distributions of the true predictors were generated to derive clinically meaningful values in accordance with the above true generating mechanism, the distributions of non-predictor variables were chosen with the intention to create variables with complex correlation structures and a range of different distributions.

The data generating code including the applied transformations is provided in the supplemental material. For data generation, the variance *σ*^2^ of the random error was set to 2 resulting in a *R*^2^ of about 0.75. This seemed to represent a plausible situation where still some variance is present. We considered the following specific simulation settings: ∙ Preceding studies with equal sample sizes

*n*=*n*_1_=*n*_2_=*n*_3_, where *n*∈{200,500,2000}. ∙ In addition, we considered seven scenarios where at least two preceding studies show unequal sample sizes, where *n*_1_,*n*_2_,*n*_3_∈{200,500,2000}.

#### Step 2: variable selection

Within each preceding study data set, variable selection was now performed to identify the respective predictors. We thereby relied on the following two variable selection techniques known to be often applied in applications: ∙ Univariable variable selection was considered with upper *p*-value thresholds of *α*_*in*_∈{0.05,0.2} meaning that variables which showed a *p*-value smaller or equal to *α*_*in*_ were included in the full model of the preceding study. ∙ In addition, we also considered backward elimination with the AIC as selection criterion.

Subsequently, each of the 20 candidate predictors (9 true predictors, 11 non-predictors) was considered as a “known predictor” if it was identified by only one preceding study (**rule 1**), by at least two preceding studies (**rule 2**) or by all three preceding studies (**rule 3**). The set of predictors identified by these rules within the preceding studies was then considered as the set of “known predictors” (background knowledge) for the current study.

#### Step 3: performance evaluation

As the true predictors are known, the reliability of background knowledge based on the preceding studies was then evaluated by different performance indicators. The following different performance measures were investigated, where we focused on correct predictor identification (descriptive aim) and prediction performance (predictive aim). A discussion of suitable performance measures can also be found in [19]. ∙ First, we evaluated how often a specific rule to quantify background knowledge from preceding studies identified all and only the true predictors, referred to as “model selection frequency” (MSF) [12]. The rates were calculated as relative frequencies over all 10,000 random replications. A value of 1 indicates that a rule is perfectly able to identify all true predictors. ∙ Second, we also evaluated the average relative frequency for each rule resulting in a correct identification of the true predictors referred to as “true positive rate” (TPR). Here, the identified predictor set might include additional variables with a zero effect. The rates were again calculated as relative frequencies over all random replications. The TPR is always at least as high as the MSF. A value of 1 indicates again an ideal performance. ∙ Third, we calculated the average false positive and false negative rates (FPR, FNR), also denoted as type I and II errors as defined in [19]. In each random replication, the number of falsely selected non-predictor variables divided by the true number of non-predictor variables (here 11) and number of falsely not-selected true predictors divided by the number of true predictor variables (here 9) were evaluated. Both numbers were then averaged over all random replications to give the FPR and FNR for a scenario, respectively. Values of 0 indicate a perfect performance. ∙ Fourth, we calculated the descriptive model selection frequency (DMSF), defined as the average relative frequency over all iterations of models, which selected the five most important predictors according to standardized regression coefficients. Considering our data generating mechanism, those five variables were *X*_*sex*_,*X*_*age*_,*X*_*cbp*.*first*_,*X*_*antihyp*_ and *X*_*cbp*.*change*_. Again, a value of 1 indicates perfect selection of the five most important predictors. For a model with a descriptive aim, the DMSF defines a natural performance indicator. ∙ Finally, we calculated the average mean square prediction error (MSPE) as the average over all simulation runs over the mean of the squared differences from the predicted and the observed outcome on a simulated data set as proposed in [19]. Therefore, we first generated a current data set of size *n*=500 for estimation of the regression coefficients which defines the proposed model. Then, we generated a validating data set and performed prediction using the proposed model. This prediction is then compared to the true outcome of the validating data set, which gives the MSPE. This procedure is repeated to define the average MSPE. A value of 0 indicates a perfect prediction. The MSPE is not bounded from above, but its value can be used to compare different models. This performance measure naturally captures the view of a predictive model, whereas for a purely descriptive model the MSPE is less important.

The investigated scenarios resulting from different simulation and analysis combinations are characterized by 1) the sample sizes of the three simulated preceding studies, 2) the variable selection technique applied for the preceding studies and 3) the selection criteria and threshold (*p*-value or AIC). As we simulated three scenarios with equal sample sizes for the preceding studies and seven with unequal sample sizes, which could then all be combined with either univariable selection (considered *p*-value threshold of 0.05 or 0.2) or backward elimination (AIC), this resulted in a total of 30 scenarios listed in detail in Table 1. The simulations were implemented in R Version 3.5 with 10’000 random replications for each setting and a seed of 29112018 to assure reproducibility.

**Table 1:** Investigated simulation scenarios

Scenario	Sample size in prior			Variable selection used in prior study
	study			
	*n*_1_	*n*_2_	*n*_3_	
S1_a	200	200	200	Univariable selection with p=0.05
S1_b				Univariable selection with p=0.2
S1_c				Backward Elimination with AIC
S2_a	500	500	500	Univariable selection with p=0.05
S2_b				Univariable selection with p=0.2
S2_c				Backward Elimination with AIC
S3_a	2000	2000	2000	Univariable selection with p=0.05
S3_b				Univariable selection with p=0.2
S3_c				Backward Elimination with AIC
S4_a	200	200	500	Univariable selection with p=0.05
S4_b				Univariable selection with p=0.2
S4_c				Backward Elimination with AIC
S5_a	200	500	500	Univariable selection with p=0.05
S5_b				Univariable selection with p=0.2
S5_c				Backward Elimination with AIC
S6_a	200	500	2000	Univariable selection with p=0.05
S6_b				Univariable selection with p=0.2
S6_c				Backward Elimination with AIC
S7_a	200	200	2000	Univariable selection with p=0.05
S7_b				Univariable selection with p=0.2
S7_c				Backward Elimination with AIC
S8_a	500	500	2000	Univariable selection with p=0.05
S8_b				Univariable selection with p=0.2
S8_c				Backward Elimination with AIC
S9_a	200	2000	2000	Univariable selection with p=0.05
S9_b				Univariable selection with p=0.2
S9_c				Backward Elimination with AIC
S10_a	500	2000	2000	Univariable selection with p=0.05
S10_b				Univariable selection with p=0.2
S10_c				Backward Elimination with AIC

## Results

Table 2 shows the resulting performance measures for the 30 selected scenarios presented in Table 1.

**Table 2:** Performance measures for investigated simulation scenarios

Scenario	S(*)_a: Univariable selection,				S(*)_b: Univariable selection,				S(*)_c: Backward elimination,			
	*α*_*in*_**=0.05**				*α*_*in*_**=0.2**				AIC			
	MSF	TPR	FPR	FNR	MSF	TPR	FPR	FNR	MSF	TPR	FPR	FNR
S1_(*)	R1: 0	R1: 0.247	R1: 0.280	R1: 0.091	R1: 0	R1: 0.644	R1: 0.607	R1: 0.040	R1: 0.001	R1: 0.325	R1: 0.484	R1: 0.096
	R2: 0.001	R2: 0.014	R2: 0.092	R2: 0.182	R2: 0.001	R2: 0.189	R2: 0.232	R2: 0.101	R2: 0.006	R2: 0.021	R2: 0.102	R2: 0.218
	R3: 0	R3: 0	R3: 0.036	R3: 0.348	R3: 0.001	R3: 0.008	R3: 0.083	R3: 0.215	R3: 0	R3: 0	R3: 0.008	R3: 0.325
S2_(*)	R1: 0	R1: 0.448	R1: 0.364	R1: 0.061	R1: 0	R1: 0.805	R1: 0.681	R1: 0.022	R1: 0.001	R1: 0.422	R1: 0.452	R1: 0.070
	R2: 0	R2: 0.087	R2: 0.137	R2: 0.102	R2: 0	R2: 0.382	R2: 0.309	R2: 0.069	R2: 0.017	R2: 0.052	R2: 0.089	R2: 0.152
	R3: 0	R3: 0.006	R3: 0.088	R3: 0.135	R3: 0.001	R3: 0.067	R3: 0.129	R3: 0.108	R3: 0.001	R3: 0.001	R3: 0.006	R3: 0.233
S3_(*)	R1: 0	R1: 0.912	R1: 0.571	R1: 0.010	R1: 0	R1: 0.992	R1: 0.793	R1: 0.001	R1: 0.001	R1: 0.467	R1: 0.436	R1: 0.059
	R2: 0	R2: 0.581	R2: 0.331	R2: 0.047	R2: 0	R2: 0.889	R2: 0.531	R2: 0.012	R2: 0.034	R2: 0.092	R2: 0.081	R2: 0.102
	R3: 0	R3: 0.170	R3: 0.175	R3: 0.092	R3: 0	R3: 0.505	R3: 0.299	R3: 0.055	R3: 0.007	R3: 0.007	R3: 0.005	R3: 0.126
S4_(*)	R1: 0	R1: 0.337	R1: 0.312	R1: 0.074	R1: 0	R1: 0.711	R1: 0.635	R1: 0.032	R1: 0.001	R1: 0.378	R1: 0.476	R1: 0.083
	R2: 0.001	R2: 0.031	R2: 0.108	R2: 0.142	R2: 0	R2: 0.244	R2: 0.256	R2: 0.088	R2: 0.010	R2: 0.029	R2: 0.098	R2: 0.193
	R3: 0	R3: 0	R3: 0.049	R3: 0.298	R3: 0.003	R3: 0.018	R3: 0.095	R3: 0.181	R3: 0	R3: 0	R3: 0.008	R3: 0.303
S5_(*)	R1: 0	R1: 0.401	R1: 0.340	R1: 0.067	R1: 0	R1: 0.764	R1: 0.659	R1: 0.026	R1: 0.001	R1: 0.405	R1: 0.462	R1: 0.075
	R2: 0.001	R2: 0.061	R2: 0.125	R2: 0.110	R2: 0	R2: 0.322	R2: 0.285	R2: 0.076	R2: 0.014	R2: 0.044	R2: 0.093	R2: 0.168
	R3: 0	R3: 0.001	R3: 0.065	R3: 0.231	R3: 0.003	R3: 0.037	R3: 0.110	R3: 0.148	R3: 0	R3: 0	R3: 0.007	R3: 0.273
S6_(*)	R1: 0	R1: 0.678	R1: 0.456	R1: 0.036	R1: 0	R1: 0.917	R1: 0.734	R1: 0.009	R1: 0.001	R1: 0.458	R1: 0.461	R1: 0.061
	R2: 0	R2: 0.157	R2: 0.166	R2: 0.096	R2: 0	R2: 0.488	R2: 0.359	R2: 0.057	R2: 0.023	R2: 0.063	R2: 0.092	R2: 0.141
	R3: 0.001	R3: 0.003	R3: 0.071	R3: 0.222	R3: 0.002	R3: 0.068	R3: 0.131	R3: 0.141	R3: 0.001	R3: 0.001	R3: 0.006	R3: 0.258
S7_(*)	R1: 0	R1: 0.644	R1: 0.440	R1: 0.040	R1: 0	R1: 0.899	R1: 0.720	R1: 0.011	R1: 0.001	R1: 0.465	R1: 0.469	R1: 0.061
	R2: 0.003	R2: 0.080	R2: 0.139	R2: 0.130	R2: 0	R2: 0.403	R2: 0.322	R2: 0.069	R2: 0.015	R2: 0.051	R2: 0.096	R2: 0.167
	R3: 0	R3: 0.001	R3: 0.051	R3: 0.295	R3: 0.004	R3: 0.036	R3: 0.110	R3: 0.177	R3: 0	R3: 0.001	R3: 0.007	R3: 0.292
S8_(*)	R1: 0	R1: 0.705	R1: 0.467	R1: 0.033	R1: 0	R1: 0.933	R1: 0.740	R1: 0.007	R1: 0.001	R1: 0.453	R1: 0.448	R1: 0.062
	R2: 0	R2: 0.199	R2: 0.188	R2: 0.089	R2: 0	R2: 0.557	R2: 0.387	R2: 0.049	R2: 0.023	R2: 0.070	R2: 0.087	R2: 0.129
	R3: 0.001	R3: 0.016	R3: 0.098	R3: 0.126	R3: 0.001	R3: 0.136	R3: 0.159	R3: 0.098	R3: 0.002	R3: 0.002	R3: 0.006	R3: 0.211
S9_(*)	R1: 0	R1: 0.834	R1: 0.523	R1: 0.019	R1: 0	R1: 0.970	R1: 0.767	R1: 0.003	R1: 0.001	R1: 0.475	R1: 0.454	R1: 0.058
	R2: 0	R2: 0.362	R2: 0.252	R2: 0.071	R2: 0	R2: 0.729	R2: 0.455	R2: 0.030	R2: 0.031	R2: 0.090	R2: 0.088	R2: 0.108
	R3: 0.002	R3: 0.011	R3: 0.082	R3: 0.213	R3: 0.003	R3: 0.128	R3: 0.166	R3: 0.130	R3: 0.001	R3: 0.001	R3: 0.006	R3: 0.237
S10_(*)	R1: 0	R1: 0.840	R1: 0.531	R1: 0.018	R1: 0	R1: 0.977	R1: 0.773	R1: 0.003	R1: 0.001	R1: 0.464	R1: 0.446	R1: 0.060
	R2: 0	R2: 0.403	R2: 0.267	R2: 0.066	R2: 0	R2: 0.774	R2: 0.472	R2: 0.025	R2: 0.031	R2: 0.091	R2: 0.085	R2: 0.106
	R3: 0.001	R3: 0.048	R3: 0.119	R3: 0.114	R3: 0.001	R3: 0.264	R3: 0.210	R3: 0.083	R3: 0.003	R3: 0.003	R3: 0.006	R3: 0.179

*MSF* Model selection frequency; *TPR* True positive rate; *FPR* Average false positive rate; *FNR* Average false negative rate; *R1* Rule 1 (one out of three preceding studies identifies predictor); *R2* Rule 2 (Two out of three preceding studies identify predictor); *R3* Rule 3 (all three preceding studies identify predictor)

It can be seen that independently of the scenario, the true predictor set was hardly ever selected with model selection frequencies (MSF) always lower than 0.005 for univariable selection and values lower than 0.04 for backward elimination (columns 2, 6, 10).

Models containing all but not only true predictors were identified more often as indicated by TPR values well above MSF (columns 3, 7, 11). In the comparison of the different rules, rule 1 was clearly the best with respect to the TPR across all scenarios followed by rule 2 and rule 3. This is intuitive as a model that only contains predictors identified by at least one preceding study (rule 1) is more likely to contain all true predictors than if the selection is more restrictive. Across all scenarios, the univariable selection with *α*_*in*_=0.2 showed the highest TPR with ranges of 0.644 to 0.992 for the most favourable rule 1, whereas univariable selection with *α*_*in*_=0.05 led to TPR in the range between 0.247 to 0.912 and backward elimination showed the worst TPR with a range of 0.325 to 0.475 for rule 1.

In contrast, rule 1 turned out to be the worst in terms of FPR. Moreover, a higher *p*-value threshold for univariable selection resulted in increased average FPR (columns 4, 8). Backward elimination showed average FPR that were a little lower than those for univariable selection with *α*_*in*_=0.2 (columns 8, 12).

At the same time, a higher *p*-value threshold for univariable selection naturally resulted in lower FNR (columns 5, 9). For backward elimination, higher average FNR were observed than for univariable selection with *α*_*in*_=0.2 (columns 9, 13). Since for predictive models a low FNR is desirable, rule 1 and a higher *p*-value threshold for univariable selection would be preferred.

**Fig. 3:**
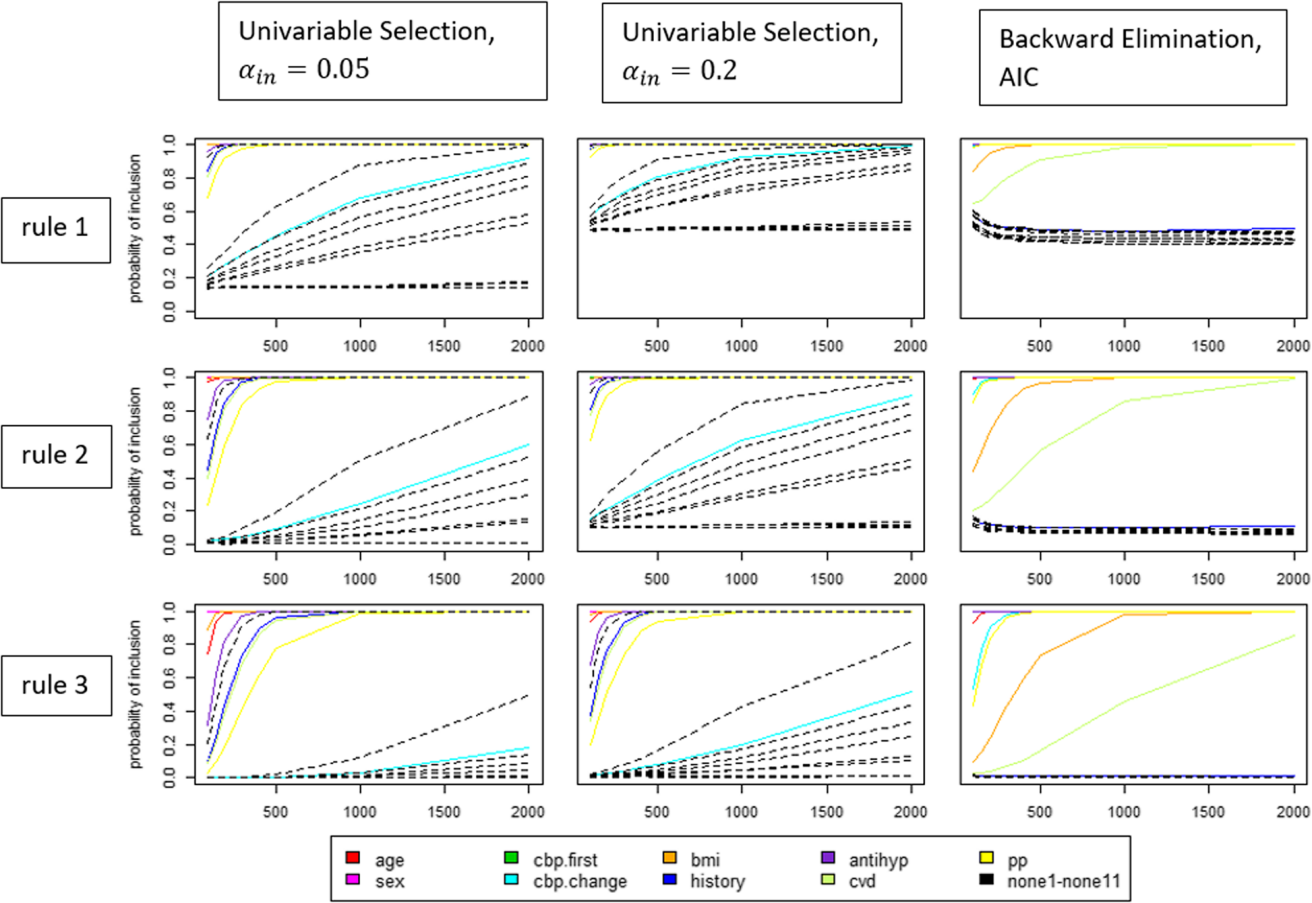
Rates of inclusion for the individual variables. Dashed, black lines refer to the non-predictors *X*_*none*1_ to *X*_*none*11_ and the solid coloured lines to the true predictors. Sample sizes for all three preceding study data sets are assumed to be equal

Whereas Table 2 provides performance measures across the complete set of candidate predictors, in Fig. 3 the rates of inclusion for the individual variables (true predictors and non-predictors) are graphically summarized as functions of the preceding study sample size (with assumed equal sample sizes in all three preceding studies).

Figure 3 shows how well the different selection methods and rules could yield a separation between the true predictors and the non-predictors. Ideally, the coloured lines (true predictors) should take values close to 1 whereas all black lines should be close to 0. Generally, it can be observed that backward elimination allowed for the best differentiation between true predictors and non-predictors, whereas the univariable selection approaches behaved similar, but a better separation was achieved with *α*_*in*_=0.05.

The univariable selection approaches tended to overlook the true predictor *X*_*cbp*.*change*_, with corresponding true regression coefficient of 0.31, whereas backward elimination did discover it. In contrast, for backward elimination *X*_*history*_ with a regression coefficient of -0.03 had the smallest inclusion frequency. Univariable selection, however, did identify it correctly. This can be explained as *X*_*history*_ was highly correlated to *X*_*sex*_, which had a high inclusion frequency. There is the general tendency that the more preceding studies were required to identify the same predictor, the better the differentiation between true predictors and non-predictors became. In this view, rule 3 was the best, followed by rule 2 and rule 1.

For a model with a descriptive aim, it is important to capture the variables that exhibit the strongest associations with the outcome in the multivariable context. This was evaluated with the DMSF reported in Table 3. Here, we identified a clear advantage of backward elimination. Across all scenarios using backward elimination the DMSF ranged from 0.895 to 1 whereas for univariable selection with an *α*_*in*_=0.05 the DMSF decreased to 0.001 for rule 3.

**Table 3:** Descriptive model selection frequencies (DMSF) for investigated simulation scenarios

Scenario	S(*)_a: Univariable selection,	S(*)_b: Univariable selection,	S(*)_c: Backward elimination,
	*α*_*in*_**=0.05**	*α*_*in*_**=0.2**	AIC
S1_(*)	R1: 0.271	R1: 0.648	R1: 1
	R2: 0.028	R2: 0.212	R2: 0.997
	R3: 0.001	R3: 0.024	R3: 0.894
S2_(*)	R1: 0.448	R1: 0.805	R1: 1
	R2: 0.086	R2: 0.382	R2: 1
	R3: 0.006	R3: 0.069	R3: 0.999
S3_(*)	R1: 0.912	R1: 0.992	R1: 1
	R2: 0.581	R2: 0.889	R2: 1
	R3: 0.170	R3: 0.505	R3: 1
S4_(*)	R1: 0.340	R1: 0.711	R1: 1
	R2: 0.044	R2: 0.255	R2: 0.999
	R3: 0.001	R3: 0.037	R3: 0.929
S5_(*)	R1: 0.401	R1: 0.764	R1: 1
	R2: 0.064	R2: 0.323	R2: 1
	R3: 0.003	R3: 0.054	R3: 0.966
S6_(*)	R1: 0.678	R1: 0.917	R1: 1
	R2: 0.161	R2: 0.489	R2: 1
	R3: 0.009	R3: 0.097	R3: 0.959
S7_(*)	R1: 0.644	R1: 0.899	R1: 1
	R2: 0.107	R2: 0.419	R2: 0.998
	R3: 0.005	R3: 0.072	R3: 0.929
S8_(*)	R1: 0.705	R1: 0.933	R1: 1
	R2: 0.200	R2: 0.557	R2: 1
	R3: 0.019	R3: 0.140	R3: 1
S9_(*)	R1: 0.834	R1: 0.970	R1: 1
	R2: 0.362	R2: 0.729	R2: 1
	R3: 0.029	R3: 0.184	R3: 0.965
S10_(*)	R1: 0.840	R1: 0.977	R1: 1
	R2: 0.403	R2: 0.774	R2: 1
	R3: 0.052	R3: 0.267	R3: 1

*R1* Rule 1 (one out of three preceding studies identifies predictor); *R2* Rule 2 (Two out of three preceding studies identify predictor); *R3* Rule 3 (all three preceding studies identify predictor)

In order to evaluate the predictive performance of the models, we report the MSPE in Table 4. The only clear result is that the MSPE is always the lowest for rule 1 followed by rule 2 and rule 3 in all scenarios. This is in line with expectations as rule 1 naturally selects models with larger numbers of predictors than rules 2 and 3. The MSPE does not generally decrease with sample size of the preceding studies, which is due to the fact that for the current data set a fixed sample size of 500 was used. Moreover, there is no clear advantage of any selection procedure used in the preceding studies.

**Table 4:** Mean square prediction error for investigated simulation scenarios

Scenario	S(*)_a: Univariable selection,	S(*)_b: Univariable selection,	S(*)_c: Backward elimination,
	*α*_*in*_**=0.05**	*α*_*in*_**=0.2**	AIC
S1_(*)	R1: 4.521	R1: 4.173	R1: 4.293
	R2: 4.669	R2: 4.362	R2: 4.250
	R3: 4.933	R3: 4.536	R3: 4.307
S2_(*)	R1: 4.252	R1: 4.151	R1: 4.227
	R2: 4.349	R2: 4.147	R2: 4.186
	R3: 4.401	R3: 4.183	R3: 4.231
S3_(*)	R1: 4.088	R1: 4.466	R1: 4.278
	R2: 4.157	R2: 4.503	R2: 4.244
	R3: 4.265	R3: 4.683	R3: 4.239
S4_(*)	R1: 4.102	R1: 4.133	R1: 3.942
	R2: 4.261	R2: 4.320	R2: 3.966
	R3: 4.604	R3: 4.485	R3: 4.038
S5_(*)	R1: 4.319	R1: 4.144	R1: 4.231
	R2: 4.409	R2: 4.258	R2: 4.198
	R3: 4.686	R3: 4.419	R3: 4.297
S6_(*)	R1: 4.460	R1: 4.133	R1: 4.000
	R2: 4.479	R2: 4.162	R2: 4.335
	R3: 4.727	R3: 4.303	R3: 4.377
S7_(*)	R1: 4.342	R1: 4.111	R1: 4.223
	R2: 4.665	R2: 4.215	R2: 4.234
	R3: 4.993	R3: 4.429	R3: 4.325
S8_(*)	R1: 4.127	R1: 4.864	R1: 4.083
	R2: 4.251	R2: 4.965	R2: 4.089
	R3: 4.429	R3: 5.119	R3: 4.113
S9_(*)	R1: 4.074	R1: 4.619	R1: 4.012
	R2: 4.167	R2: 4.617	R2: 3.998
	R3: 4.369	R3: 4.767	R3: 4.061
S10_(*)	R1: 4.058	R1: 4.047	R1: 4.018
	R2: 4.142	R2: 4.115	R2: 3.991
	R3: 4.212	R3: 4.337	R3: 4.009

*R1* Rule 1 (one out of three preceding studies identifies predictor); *R2* Rule 2 (Two out of three preceding studies identify predictor); *R3* Rule 3 (all three preceding studies identify predictor)

Still, for reasonable preceding study sample sizes of 500, the probabilities of inclusion for the true predictors were often considerably below 1 for all rules and selection techniques. In addition, the probabilities of inclusion for the non-predictors were mostly considerably above 0 for all rules combined with univariable selection.

## Discussion

The results of our simulation study suggest that the variable selection techniques used in preceding studies may have major effects on the level of evidence for what is called background knowledge. In our study, we investigated how inappropriate selection methods in such preceding studies can translate into poor representation of background knowledge in a given study. The simulation study showed that when the commonly applied univariable selection was used, the identified set of variables might not be reliable, even when several preceding studies have identified the same predictor. The stability of the identified predictor set is also limited if a more appropriate selection method such as backward elimination has been applied. Our results showed that choosing only variables which have been pre-identified multiple times does not necessarily improve the rates of correct inclusion of true predictors in general, but only reduces the rate of wrong inclusion for non-predictors. Moreover, our results show that the predictive performance of the resulting models is also limited independent of the variable selection procedure in the preceding studies.

The identification of true predictors by one or several preceding studies also depends on the underlying sample size and the number of candidate predictors. In the literature, the ratio of sample size and candidate variables is assessed via the ratio of “events per variable” (EPV). A rule of thumb says that at least 10 observations are required per variable in linear regression models [20]. This implies that for the situation considered in here with 20 candidate variables, the sample size should be at least 200. However, with preceding studies of that size, results were not yet satisfactory in our simulation study. Sometimes even a recommendation of at least 50 EPV is given, which is better in line with our results [21]. Recent development even goes one step further, Riley et al. (2020) state that the calculation of the sample size should also incorporate other factors like the expected predictive performance of the model [22]. Here, results improved only if very high sample sizes of 1000 or more were assumed, which in some practical situations may not be achievable.

Thus, results shown here are limited for various reasons. A very general point of criticism regarding simulation studies is the assumption of the existence of a true underlying model. Several authors already declared that they do not agree with this assumption [2, 21, 23]. Nonetheless, when evaluating the performance of a model, the assumption of different data generating mechanisms helps in understanding and comparing properties of the evaluated model building strategies. Despite analysing a broad variety of scenarios, simulation studies are never able to cover all possible settings eventually found in applications. In here, the same simulation design was applied for all three preceding data sets, whereas changes in the correlation structure and the variable distributions might have an impact.

Further, the relative amount of non-predictors impacts the performance. We also investigated different settings with fewer non-predictors referring to a lower signal-to-noise ratio (results not shown). Even in this setting, where it should be easier to identify the true predictors, the performance measures indicated no relevant improvement compared to the scenarios shown here.

In this work, we concentrated on a multivariable linear regression model. For future research, other regression model types with a nonlinear link function (logistic, Cox regression) implying noncollapsibility issues could be investigated. We assume that with such models, selection uncertainty is even higher.

We have focused on background knowledge from a black-or-white perspective, that is a predictor is either included or not. Incorporating the uncertainty of background knowledge could most naturally be done by using Bayesian hierarchical models, as e.g. done by Gracia et al. (2015) [24] or using informative prior distributions in an empirical Bayes approach [25]. Unfortunately, those methods are rarely used in clinical research. As the aim of our work was to assess the impact of commonly applied methods, Bayesian methods were not further investigated although they are worthwhile to be considered in applications.

As a further limitation, we only considered low dimensional data. Generally, methodological results on model building for low and high dimensional data might deviate [26] so that our results cannot be transferred easily to the situation of high dimensional data. A similar investigation of background knowledge in variable selection for high-dimensional omics data was conducted by Liu et al. [27]. They proposed to integrate background knowledge along with marginal correlations in the prescreening procedure of omics data before applying the LASSO. A similar method was proposed by Bergersen et al. [28] who directly integrated background knowledge by weighting the penalties of each regression coefficient.

Note that within this work, we were interested in identifying the correct set of predictors and/or a good prediction which is important in the context of descriptive and predictive models. We did not focus on explanatory models and therefore did not assess the accuracy in estimation of the regression coefficients. However, one interesting aspect would be to look at the common recommendation to control for all variables that are causes of either the exposure or the outcome and not on the causal pathway [29]. Nevertheless, poor methodology in preceding studies may also increase the risk to not correctly identify and specify confounders in an explanatory study.

## Conclusion

To conclude, we strongly advised to identify the source and the level of evidence for so called “background knowledge”. If background knowledge is only based on a few preceding studies without sufficient biological support, the methodology of these studies should be carefully investigated, and uncertainties related to the selection or non-selection of variables in such studies should be critically inferred [12]. This does not imply a recommendation against the incorporation of background knowledge in model building, but rather aims at making researchers more sensitive to a critical appraisal of the existing evidence.

## Supplementary Information

**Additional file 1S 1:** Average correlation coefficients for the resulting transformed candidate predictors. **S 2:** Data simulating R code.

## Data Availability

No original trial data are used in this work. Simulated data and software source code that support the findings of the simulation study can be found in the supplemental material.
