# Supplementary figures and images for: Statistical model building: Background “knowledge” based on inappropriate preselection causes misspecification
Source: BMC Med Res Methodol. 2021 Sep 29;21:196. doi: 10.1186/s12874-021-01373-z (PMC8480029; doi:10.1186/s12874-021-01373-z)

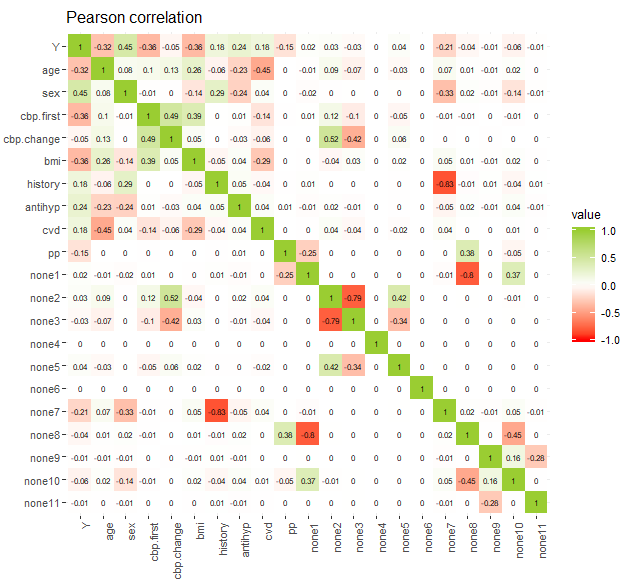

Supplement: Supplementary file 1 — Additional file 1S 1: Average correlation coefficients for the resulting transformed candidate predictors. S 2: Data simulating R code. [file 12874_2021_1373_MOESM1_ESM.zip › 12874_2021_1373_MOESM1_ESM/FigureS1.PNG]
